# Development of the Pulmonary Vein and the Systemic Venous Sinus: An Interactive 3D Overview

**DOI:** 10.1371/journal.pone.0022055

**Published:** 2011-07-11

**Authors:** Gert van den Berg, Antoon F. M. Moorman

**Affiliations:** Department of Anatomy, Embryology and Physiology, Academic Medical Center, Heart Failure Research Center, Amsterdam, The Netherlands; Brigham & Women's Hospital - Harvard Medical School, United States of America

## Abstract

Knowledge of the normal formation of the heart is crucial for the understanding of cardiac pathologies and congenital malformations. The understanding of early cardiac development, however, is complicated because it is inseparably associated with other developmental processes such as embryonic folding, formation of the coelomic cavity, and vascular development. Because of this, it is necessary to integrate morphological and experimental analyses. Morphological insights, however, are limited by the difficulty in communication of complex 3D-processes. Most controversies, in consequence, result from differences in interpretation, rather than observation. An example of such a continuing debate is the development of the pulmonary vein and the systemic venous sinus, or “sinus venosus”. To facilitate understanding, we present a 3D study of the developing venous pole in the chicken embryo, showing our results in a novel interactive fashion, which permits the reader to form an independent opinion. We clarify how the pulmonary vein separates from a greater vascular plexus within the splanchnic mesoderm. The systemic venous sinus, in contrast, develops at the junction between the splanchnic and somatic mesoderm. We discuss our model with respect to normal formation of the heart, congenital cardiac malformations, and the phylogeny of the venous tributaries.

## Introduction

Early cardiac development is inseparably associated with the formation of the embryonic venous systems. Initially, the embryonic vasculature consists only of bilaterally paired vitelline veins, located between the endoderm of the yolk sac and the splanchnic mesoderm. These vitelline veins fuse in the embryonic midline, forming a ventral vessel [1], [2]. The primary heart tube is formed as the walls of this vessel differentiate into myocardium [3] (review: [4]). With subsequent growth of the embryo proper, there is development of the cardinal and the umbilical venous systems. In those species relying on lungs for the oxygenation of blood, there is also formation of a pulmonary vein or veins. During the development of these vessels, and their connection to the heart, the myocardium grows and changes shape by a combination of proliferation [5], [6], and addition and muscularization of precursor cells [7]–[10]. Many malformations of the cardiac inflow have their origin in this early developmental period, during which numerous processes intertwine both spatially and temporally. These abnormalities range from simple deformities, such as persisting communications across the oval foramen, to complex malformations involving abnormal drainage of the pulmonary vein to extra-cardiac sites [11].

Despite this obvious clinical relevance, the long-established morphological examination of the development of the inflow has yet to produce consensus, as illustrated by the ongoing discussion regarding the relation between development of the pulmonary vein and the systemic venous tributaries [12], [13]. The reasons for such ongoing disputes lie in part in the lack of use of molecular markers to distinguish between cell types [14], but also reflect the difficulty of communicating complex three-dimensional processes. Together, these ongoing problems prevent the emergence of a clear morphological concept of development of the venous inflows that could serve as a scaffold for further study. To provide such morphological insights, we conducted a three dimensional study of the developing venous pole of the heart in the chick, using specific markers for myocardium and working myocardium of the chambers. We have used chicken embryos as our experimental model because of the prominence of the vitelline veins, and the flatness of the blastoderm. These features result in a reduced complexity of general embryonic morphology, and are shared with human embryos. The resulting reconstructions are presented via a novel interactive 3D-technique (File S1).

The reconstructions show how the pulmonary vein develops by separation from a greater venous plexus, which is located within the splanchnic mesoderm. The systemic venous tributaries, in contrast, develop laterally on the junction between the splanchnic and somatic mesoderm by muscularization of the mesenchyme that surrounds the common cardinal veins. We show how this model of development can explain the known clinical variability of abnormal pulmonary venous return. Using this model, we also discuss the origin of the pulmonary circulation from a phylogenetic perspective. Although our proposed model of development is not novel in itself, we submit that our three-dimensional and interactive presentation will help in resolving the debate about the formation of the systemic and pulmonary venous tributaries.

### Background to concepts of development of the venous inflow

**Figure 1:**
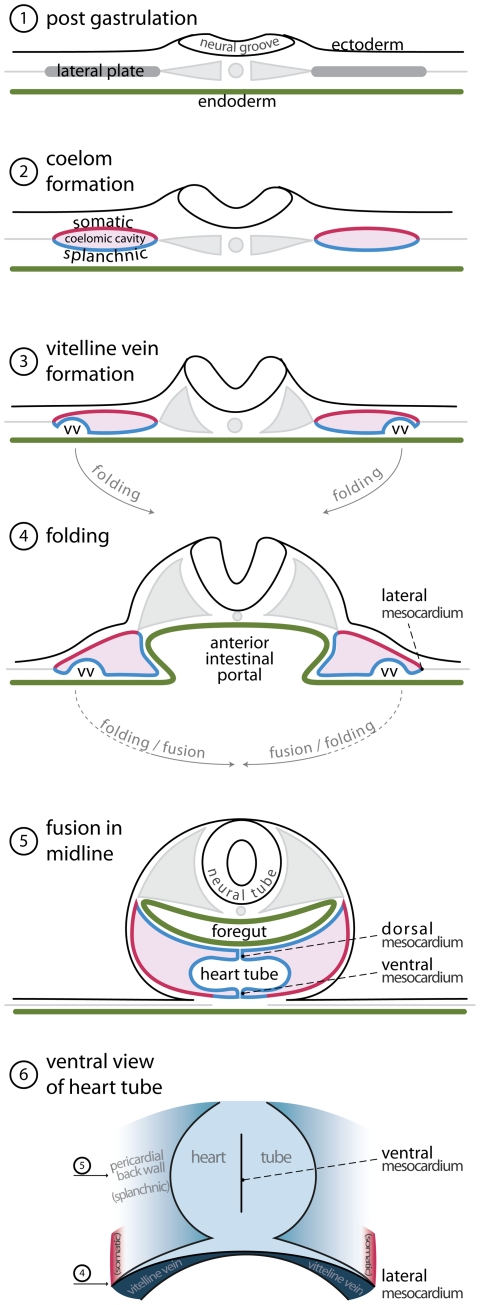
Transition of trilaminar embryonic disc during early development. (**1**) Trilaminalar embryonic disc after gastrulation; lateral plate mesoderm resides between the endoderm (green) and the ectoderm (black). (**2**) The lateral plate mesoderm separates in a splanchnic (blue) and a somatic (red) layer by the formation of the coelomic cavity (violet). (**3**) The lateral edges of the splanchnic mesoderm luminize, forming the bilateral vitelline veins. (**4**) By folding the lateral edges of the embryonic disc move inwards, creating the anterior intestinal portal. The persisting contact between the somatic and splanchnic mesoderm is called the lateral mesocardium. (**5**) Fusion of the bilateral vitelline veins generates the primary heart tube. The point of fusion is recognized as the ventral mesocardium. The medial splanchnic mesoderm becomes the pericardial back wall, which is connected to the heart tube via the dorsal mesocardium. (**6**) Ventral view of the primary heart tube. The height of cross sections (4) and (5) is indicated by arrows. (Abbreviation–vv: vitelline vein.)

Prior to presentation of our observations, we firstly discuss existing studies regarding formation of the cardiac venous inflow. Classical cardiac embryologists had a profound knowledge of early cardiac development. A great deal of these publications is now relatively obscure, but remains of significance for current-day research. Figure 1 offers an overview of classic and current knowledge regarding the formation of the venous inflows.

After gastrulation, the embryo can be represented as a trilaminar disc made up of endoderm, mesoderm, and ectoderm. The heart tube and its venous tributaries, are derivatives of the mesoderm. With formation of the coelomic cavity, lateral plate mesoderm separates into splanchnic and somatic layers, which line the endoderm and the ectoderm, respectively. The lateral edges of the splanchnic mesoderm gradually lumenize to form the vitelline veins. These veins basically are bilateral gutters of splanchnic epithelia that both contain an endothelial tube. These endothelial tubes are caudally contiguous with the blood islands that reside in the extra-embryonic mesoderm covering the yolk sac. As the embryo folds, the paired vitelline veins progressively fuse in the embryonic midline. The walls of these fused veins, facing the future pericardial cavity, initiate expression of cardiac sarcomeric proteins [2], [15], and, shortly following fusion, start to contract rhythmically [16], thus forming the so-called primary heart tube (review: [4]). It needs to be noted that the walls of these vitelline veins do not proliferate, and that the heart tube is gradually formed by a progressive addition of rapidly dividing cells from bordering splanchnic mesoderm [9]. The vitelline veins continue to fuse subsequent to formation of the heart tube, becoming the portal vein. Together with the umbilical veins, the portal vein contributes to the hepatic vascular bed [17], [18].

Because the primary myocardial heart tube forms by the fusion of two epithelial gutters, it is initially unclosed dorsally, where the so-called dorsal mesocardium connects the heart to the splanchnic mesoderm that overlies the embryonic pharynx. This mesocardium is the only site through which vessels or additional tissue can dorsally enter the heart (Figure 1, panel 5). Prior to folding, this “pharyngeal” mesoderm was located medially to the lumenizing mesoderm of the vitelline veins. Upon fusion of the left and right coelomic cavity, the mesoderm which was originally located laterally to the vitelline veins will form the so-called ventral mesocardium, a transient structure that disintegrates shortly after the fusion of the vitelline veins [2]. At this point in development, the functional embryonic circulatory system is comparable to that of primitive organisms [19]. Blood from the blood islands is transported through the embryo by a heart that is nothing more than a sluggishly contracting ventral vessel that overlies an endodermal gut. Subsequent to this developmental stage, the other systemic venous systems, namely the cardinal and umbilical veins, develop, as further explained below.

At the level of the anterior intestinal portal, the ventral mesocardium is contiguous with the lateral mesocardial connections. These lateral connections, although rarely discussed in literature, form an important landmark in embryonic development, as they reflect the contact between the somatic and splanchnic layers of the mesoderm [18], [20]. Besides playing an important role in separating the embryonic coelomic cavity, these lateral mesocardial connections also envelop the forming common cardinal veins [18]. These latter veins, lying in somatic mesoderm [20], facilitate the venous drainage from the embryo proper. They receive blood from the superior and inferior cardinal veins, as well as the umbilical veins, which run in the lateral body wall. In chicken embryos, the umbilical veins return blood from the allantois. Eventually, a discrete “sinus venosus”, or systemic venous sinus, is formed by myocardial differentiation of the mesenchyme that surrounds the common cardinal veins, and by incorporation of these structures into the pericardial cavity [10], [21]. In both chicken and mouse embryos, this mesenchyme expresses *Tbx18*, and is devoid of *Nkx2–5* expression. Knock-out of the Tbx18 gene in mouse disrupts both formation of the sinus horns and separation of the coelomic cavity [21].

The fourth venous system that becomes connected to the cardiac inflow is the one that drains the developing lungs. As already discussed, the relation between the pulmonary vein and the systemic venous tributaries has been subject to longstanding debate, specifically as to whether the pulmonary vein becomes connected to the left atrium directly (review: [22]), or via the tributaries of the sinus venosus (review: [23]). Although at first glance semantic, the consequences of these different views are very relevant with respect to the etiology of cardiac disease. If the pulmonary vein did originate from the systemic venous sinus, the pulmonary myocardium would share its lineage with myocardium that forms the sinus node, a view [23] used to explain the frequent occurrence of arrhythmias that originate from the pulmonary orifices in the left atrium [24]. Although recent lineage analyses convincingly demonstrated a distinct origin of the myocardium of the systemic and pulmonary venous tributaries [21], opposing views are still put forward [12]. Since clarification of the spatial intricacies of the development of these regions of venous inflow might resolve these differences, we undertook our 3D-analysis of this clinically important region.

## Methods

### Experimental Animal Handling and In Situ Hybridisation

According to Dutch law (“Wet op de dierproeven”), embryos are not covered by the definition of experimental animals until a viable age is reached. In chicken, this occurs at around 21–23 days of development. Our study did not include chicken embryos older than 5 days of development. Therefore, approval of an ethic committee was not necessary. Chicken embryos were acquired by timed incubation of fertilized eggs, obtained from a local hatchery. After isolation, the embryos were staged according to the system proposed by Hamburger and Hamilton [25], fixed in 4% PFA, dehydrated with a series of graded alcohol, cleared with butanol, and embedded in paraplast. After embedding, the embryos were sectioned at either 12 or 14 µm. Using in situ hybridization (ISH) [26], serial sections were sequentially stained for the presence of mRNA of cardiac Troponin I (*cTnI*) [27] and Connexin40 (*Cx40*) (GenBank no: NM_205504, kindly provided by dr. T. Mikawa).

### Image Analysis and 3D Reconstructing

**Figure 2:**
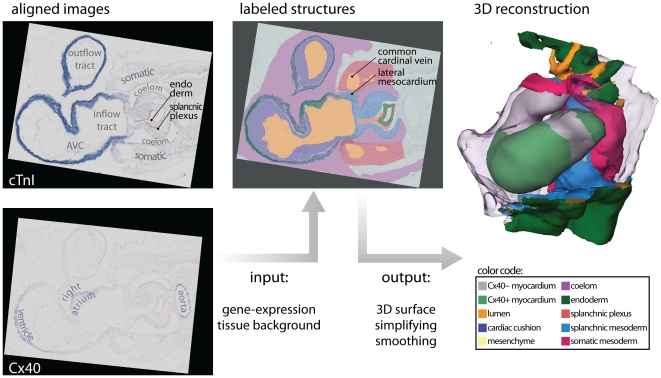
3D reconstruction from sections. The left panel shows two exemplary ISH-stains of expression of *cTnI* and *Cx40*, translated and rotated to alignment. Note that the line that indicates the splanchnic plexus and the textual annotations were later added for the sake of clarity. Using *Amira*, the signals of gene-expression and the general tissue background were transformed into a 3D reconstruction. The presented colors indicate the same structures throughout the remainder of the article. Note that yellow is used to indicate both the cardiac cushions after invasion with mesenchyme, but also for mesoderm that is of unclear splanchnic or somatic descent. (Abbreviations–3D: three dimensional; AVC: atrioventricular canal; cTnI: cardiac Troponin I; Cx40: Connexin 40.)

Digital images of the stained sections were made using bright-field illumination (Zeiss Axiophot microscope, coupled to Leica DFC321 camera). For 3D-reconstruction, the digital images were loaded into Amira (Visage Imaging, Inc.) and appointed a voxel size appropriate to section thickness and optical magnification. Images were rotated and translated to alignment. Subsequent to this alignment, the embryonic structures and the domains of gene-expression were segmented into binary labels, using general tissue contrast and specific signal from ISH-staining (Figure 2). Due to the sequential staining of the sections, the ISH-signals were interpolated over 3 sections. After inclusion of all segments, the labels were resampled to iso-volumetric voxel dimensions, and smoothed in the y and z orientations. These smoothed data-sets were transformed into a surface by triangulation. The number of triangles was then reduced to 7600, and the resulting surface itself smoothed using the SmoothSurface module of Amira. For 2D presentation, snapshots of the reconstructions were taken in Amira, and laid out and annotated using Illustrator CS4 (Adobe Systems, Inc.). For 3D-presentation, the Amira-surface files were converted into three-dimensional pdf-files with Acrobat 3D (Adobe Systems, Inc.). Using java-scripts, we prepared a custom interface for the use of the functionality of Acrobat 3D [28].

### Definitions

In order to discuss the development of the pulmonary vein with respect to the atrial chambers and the systemic venous sinus, it is imperative to define these structures accurately. The atrial myocardium is produced by local differentiation at the caudal part of the primary heart tube. A clear sign of this differentiation is the increase in the velocity of electrical conduction [29], caused by local initiation of expression of gap-junctional proteins such as Connexin40 (*Cx40*) (review: [30]). The myocardium of the systemic venous sinus is produced by muscularization of the mesenchyme that envelops the common cardinal veins. Focusing as we do here on the three-dimensional development, we define the sinus horns as the myocardium both expressing cardiac Troponin I (*cTnI*) and surrounding the common cardinal veins. Note that assigned color codes translate gradations of molecular expression into discrete values. The splanchnic and somatic mesodermal layers are separated by the coelomic cavity. The splanchnic mesoderm is the tissue between the endoderm and the coelomic cavity, while the somatic mesoderm is the tissue between the ectoderm and the coelomic cavity. The splanchnic plexus is the loosely arranged tissue inside the splanchnic mesoderm, shown previously to express a variety of vascular markers [31], [32]. We define the pulmonary vein only when it possesses a patent lumen within this plexus, running from the dorsal mesocardium to the developing lungs.

## Results

The reader is encouraged to read the results along with the interactive 3D-PDF files in the Data Supplement (File S1).

### 2 days of development-stage 12 (Figure 3, File S1 and Supplemental Figure S1)

**Figure 3:**
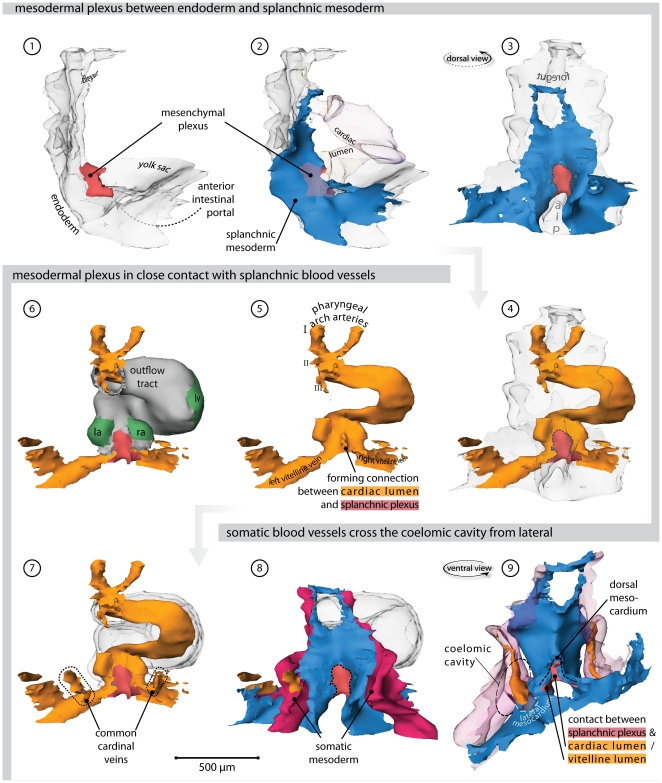
Heart region of stage 12 embryo. Also refer to supplemental File S1 for an interactive version of this reconstruction. (**1**) Right lateral view of the endoderm (transparent). Overlying the endoderm of the anterior intestinal portal is a mesenchymal plexus (red). (**2**) Overlying this mesenchymal plexus is the splanchnic mesoderm (blue). The lumen of the heart tube is transparent. (**3**) Dorsal view, showing how the vascular plexus is wedged between the endoderm and the splanchnic mesoderm. (**4**) The cardiovascular lumen is shown in orange; the forming vitelline veins are continuous with the vessels that cover the yolk sac. (**5**) Cardiac lumen protrudes into dorsal mesocardium, where contact with splanchnic plexus is being established. (**6**) Bilaterally flanking the dorsal mesocardium are the emerging atria, as distinguished by expression of Connexin40 (green). (**7**) Hooking into the vitelline veins from dorsolateral are the common cardinal veins. (**8**) These common cardinal veins reside in somatic mesoderm (red). (**9**) Ventral view of the splanchnic and somatic mesoderm. Separating these tissues is the coelomic cavity. They contact at the so-called lateral mesocardium (light blue). (Abbreviations–I, II, III: first, second & third pharyngeal arch artery, respectively; aip: anterior intestinal portal; la: left atrium; ra: right atrium; lv: left ventricle.)

Expression of Cx40 at the venous pole of the heart was first observed at stage 12, in the myocardium that laid bilaterally from the dorsal mesocardium, reflecting the differentiation of the left and right atrium from the left and right heart-forming regions, respectively [33]. At this stage, pulmonary venous structures were not observed, nor had there been formation of lungs. Instead, a loosely arranged mesenchyme, overlying the endoderm of the anterior intestinal portal and underlying the splanchnic mesoderm, was emerging. This mesenchyme, seen along the cardiovascular lumen, contacted both the lumen of the embryonic atria, via the dorsal mesocardium, and the still unfused vitelline veins. Given the absence of clear lumina in the mesenchymal plexus at these stages, drainage of the plexus on these vessels could not be morphologically assessed. As noted previously [34], it is not possible to define a sinus venosus at this stage. Rather, the recently formed common cardinal veins entered the inflow laterally, via the lateral mesocardial connections.

### 3 days of development-stage 16 (Figure 4, File S1 and Supplemental Figure S2)

**Figure 4:**
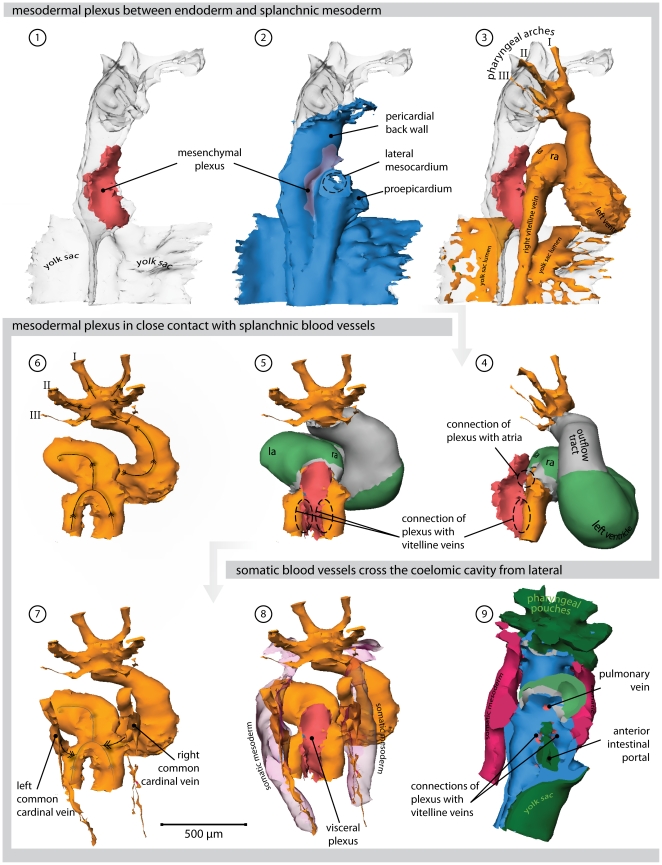
Heart region of stage 16 embryo. Also refer to supplemental File S1 for an interactive version of this reconstruction. (**1**) Right lateral view of the endoderm (transparent). Overlying the endoderm of the anterior intestinal portal is a mesenchymal plexus (red). (**2**) Overlying this plexus is the splanchnic mesoderm (blue); its connection with the somatic mesoderm, the lateral mesocardium, is removed. (**3**) Cardiovascular lumen in relation with the endoderm and splanchnic plexus. (**4**) The lumina of the blood islands covering the yolk sac are removed. Primary myocardium is shown in grey, *Cx40* positive working myocardium in green. The splanchnic plexus is separating and contacts both the atrial lumen via the dorsal mesocardium, and the vitelline veins. (**5**) Dorsal view of the plexus in relation to the myocardium and the vitelline veins. (**6**) The direction of blood-flow within the heart and vitelline vessels is indicated with arrows. (**7**) Addition of the cardinal veins. (**8**) The cardinal veins reside in somatic mesoderm (transparent red). (**9**) Ventral view, now showing the endoderm in dark green. The surface of the reconstructions is cut in a frontal plane, showing canalization of the splanchnic plexus into the cardiovascular lumen of the atria (via the dorsal mesocardium) and the vitelline veins. (Abbreviations–I, II, III: first, second & third pharyngeal arch artery, respectively; la: left atrium; ra: right atrium.)

Compared to stage 12, the domain of *Cx40* expression at the inflow had expanded, reflecting the progressive growth and differentiation of the atria. As described in mouse [21], atrial growth was more pronounced at the left side. In proximity to the dorsal mesocardium, the expression of *Cx40* and *cTnI* became weaker, reflecting the addition of newly differentiating cells from splanchnic mesoderm that covers the endoderm of the foregut. This mesoderm is currently known as the second heart-field [7]. The newly formed venous plexus, wedged between the dorsal mesocardium in the midline of the embryo, opened directly to the atrial cavity. More caudally, the remainder of the venous plexus connected with the vitelline veins. Although part of the systemic venous return, this region should not be considered to be part of the systemic venous sinus, since the latter entity has still to form at this stage. When compared to the previous stage, the common cardinal veins had enlarged, albeit remaining devoid of myocardial sleeves.

### 4 days of development-stage 21 (Figure 5, File S1 and Supplemental Figure S3)

**Figure 5:**
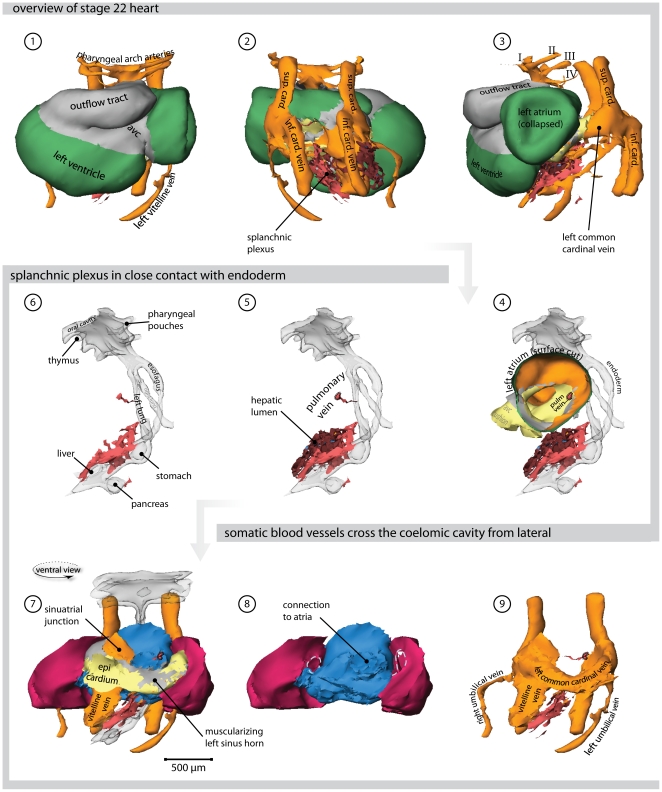
Heart region of stage 21 embryo. Also refer to supplemental File S1 for an interactive version of this reconstruction. (**1**) Ventral view of the heart. *Cx40* positive working myocardium is shown in green, primary myocardium is depicted in grey. (**2**) Dorsal view; the splanchnic plexus is shown in light red and hepatic lumen in brown. (**3**) Left lateral view. (**4**) Left lateral view; the surface of the left atrium is cut, showing the atrial septum and the entrance of the pulmonary vein. The endoderm is transparent. (**5**) The splanchnic plexus is interwoven with the hepatic lumen (**6**) Within the endoderm several organs are developing. (**7**) Ventral view showing the sinus venosus region with respect to the splanchnic (blue) and somatic (red) mesoderm (**8**) Solitary depiction of the splanchnic and the somatic mesoderm. The lateral mesocardium is indicated by a hatched line. (**9**) The venous systems of the cardiac inflow. (Abbreviations–I, II, III, IV: first, second, third & fourth pharyngeal arch artery, respectively; avc: atrioventricular canal; sup. card.: superior cardinal vein; inf. card. vein: inferior cardinal vein; pulm. vein: pulmonary vein.)

By this stage, further progression in atrial development was evident. Again, the left atrium was larger, and, in the specimen shown, its wall had collapsed during embedding (Figure 5). By now, the primary atrial septum was emerging, and the pulmonary vein could be seen to the left of this septum, having separated from the splanchnic venous plexus as a solitary vessel. The remainder of the splanchnic plexus overlaid the endoderm, which had clearly formed organs such as the stomach and the pancreas. The caudal splanchnic plexus joined with the hepatic vasculature, which was forming around the further fused vitelline veins. The entry of the systemic venous tributaries had become displaced towards the right of the embryonic midline. In consequence, the left common cardinal vein had elongated, and encircled the remainder of the splanchnic plexus. The right common cardinal vein was smaller, and was fully sheathed by myocardium. The mesenchyme surrounding the left common cardinal vein, in contrast, was only starting to become muscularized, concomitant with the onset of formation of the sinus horns.

### 5 days of development-stage 25 (Figure 6, File S1 and Supplemental Figure S4)

**Figure 6:**
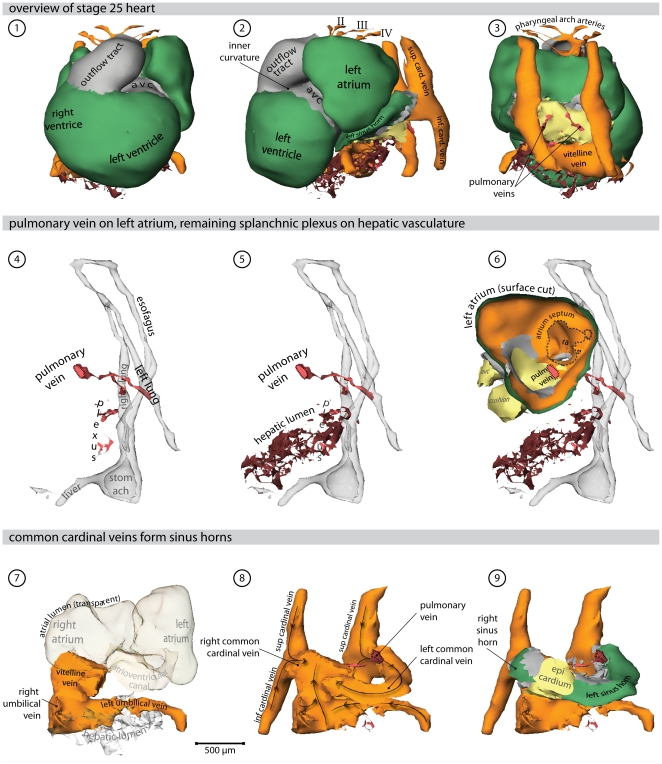
Heart region of stage 25 embryo. Also refer to supplemental File S1 for an interactive version of this reconstruction. (**1**) Ventral view of the heart. *Cx40* positive working myocardium is shown in green, primary myocardium is depicted in grey. (**2**) Left lateral view of the heart; the splanchnic plexus is shown in red and hepatic lumen in brown. (**3**) Dorsal view of the heart. (**4**) Left lateral view of the endoderm (transparent grey) in relation to the pulmonary vein and the splanchnic plexus. (**5**) The remaining plexus attaches to the hepatic lumen. (**6**) Left lateral view; the surface of the left atrium is cut, showing the atrial septum and the entrance of the pulmonary vein. (**7**) Systemic venous returns (orange) with respect to the atrial lumen (transparent). (**8**) Direction of blood-flow. (**9**) The common cardinal veins entirely ensleeved by myocardium. Most of this myocardium now expresses *Cx40*, with the exception of the forming sinus node. (Abbreviations–II, III, IV: second, third & fourth pharyngeal arch artery, respectively; avc: atrioventricular canal.)

With the addition of another day in development, the morphology of the heart had not altered significantly, despite general growth of the embryo. Due to this increased size, less endoderm was reconstructed. In the ventricles, *Cx40* was predominantly expressed in the trabecular myocardium. For the sake of clarity, however, the entire ventricular walls are presented as *Cx40*-positive. The pulmonary vein had bifurcated, and drained the endoderm of both lung buds. The primary atrial septum had also developed further, with the pulmonary vein continuing to enter the atrial cavity at its left side. By now, however, it was possible to recognize a discrete systemic venous sinus, as both common cardinal veins were fully covered by myocardium. These myocardial sleeves now expressed *Cx40*, reflecting their atrialization. It was now also possible to recognize the forming sinus node as a region devoid of *Cx40* expression within the right sinus horn, as also described for the mouse [35].

## Discussion

Using novel techniques of reconstruction and presentation, we have shown how a discrete systemic venous sinus, or “sinus venosus”, develops at the junction between the splanchnic and somatic mesoderm. The pulmonary vein, on the other hand, is shown to separate from a greater splanchnic venous plexus that extends from the heart to the liver. This model of pulmonary venous development is not novel, being supported by convincing evidence from vascular markers and injections of Indian Ink to show luminal connections [31], [32]. It is unclear, therefore, as to why this model of development has become underexposed in current literature. The concept of formation of the pulmonary vein from this greater splanchnic plexus offers useful insights into the development of the venous pole of the heart, clinical manifestations of abnormal pulmonary venous return, and sheds light on evolutionary aspects of cardiac development.

### Relation between the systemic venous sinus and the pulmonary vein

As we have discussed, the debate regarding the relation between the pulmonary vein and the sinus venosus is long-standing. Lineage analyses performed during the previous century already suggested that the common cardinal veins, which lie at the basis of future sinus horns, originate from somatic mesoderm [20]. Using the same techniques, the pulmonary vein had been shown to derive from the splanchic mesoderm that overlies the foregut [36]. Recent genetic lineage studies in mouse using the *Nkx2–5* promoter further disproved a common origin of the myocardium of the systemic venous sinus and the pulmonary vein [21]. Alternative, and more sensitive, genetic lineage suggested that the entire systemic venous sinus originated from precursors that expressed *Nkx2–5* [37]. This, however, is most probably due to early detection of recombination in the lateral plate mesoderm, not allowing the analysis of the respective lineages of the splanchnic and somatic mesoderm.

Despite this body of evidence relating to the fate and lineage of these cells, and the convincing observations of direct drainage of the pulmonary vein into the left atrium in human [38], [39], mouse [13], [40], and chicken [41], some investigators argue that the pulmonary vein originates from the sinus venosus [23], and have even introduced new cardiac components when describing normal development [12]. It is likely that this reflects differences in the interpretation of the complex 3D-architecture of the developing cardiac venous pole, resulting in different definitions of the key structures involved. Indeed, our own study shows that, during normal development, the anlage of the pulmonary vein, namely the splanchnic plexus, is connected to the developing systemic venous inlet. This does not imply, however, a relation between the pulmonary vein and the definitive systemic venous sinus. Those reaching this latter conclusion seem to have simply defined the sinus venosus as the confluence of the systemic veins. Already in 1933, however, Patten and Kramer had emphasized that such a definition can result only in “unnecessary confusion” [34]. The part of the systemic return connecting with the splanchnic plexus belongs to the future portal venous system, eventually becoming incorporated into the venous drainage of the more caudal derivatives of the gut to the liver. There is no evidence of drainage of the lumenized pulmonary vein to the definitive systemic venous sinus during normal development. Not infrequently, however, such connections are seen in a clinical situation, as we discuss below.

### Abnormal Pulmonary Venous Connections

It is well recognized that, in the congenitally malformed heart, the pulmonary venous return is through systemic venous channels rather than directly to the morphologically left atrium. The sites of such anomalous connections are diverse, and are usually divided into 1) direct right atrial connections; 2) connections through the derivatives of the right-sided common cardinal systems, namely the superior caval and azygos veins; 3) connections through derivatives of the left common cardinal system, specifically the coronary sinus; and 4) connections through the umbilicovitelline system via the portal vein and venous duct [42]. Direct drainage of the pulmonary veins to the morphologically right atrium, however, is almost always seen in the setting of isomerism of the right atrial appendages, and should not necessarily be considered as an error in separation of the splanchnic plexus.

**Figure 7:**
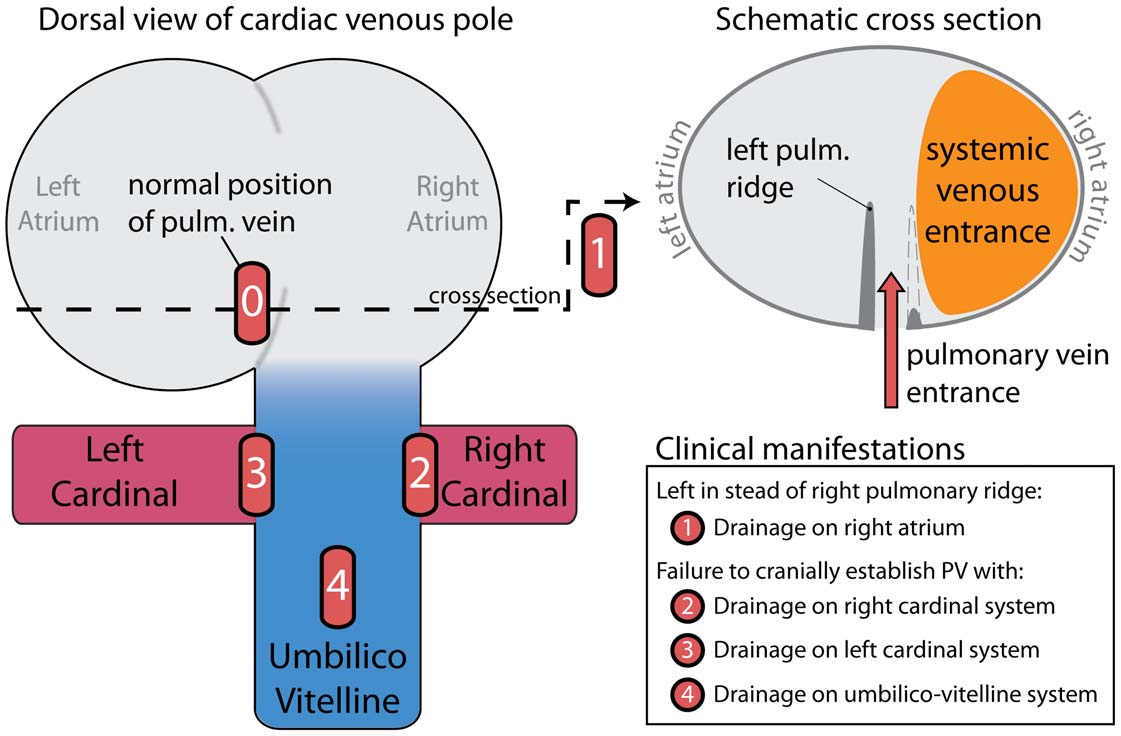
Clinical manifestations of Abnormal Pulmonary Venous Return. The left illustration shows a dorsal view of the cardiac venous pole. (**0**) Normal drainage of the pulmonary vein into the left atrium, caused by cranial separation of the pulmonary vein from the splanchnic plexus and formation of the atrium septum from the right pulmonary ridge. (**1**) In case of isomerism of the right atrial appendages, the atrium septum can form from the left pulmonary ridge. If this is the case, then the pulmonary vein will drain into the right atrium, as shown in the schematic section on the right. This should not be considered to be an erroneous separation of the pulmonary vein from the splanchnic plexus. Failure of such a cranial establishment of the pulmonary vein from this plexus can cause a variety of clinical manifestations of abnormal pulmonary venous return, depending on the caudal location of drainage (**2**, **3**, and **4**).

Although these above-described phenotypes might appear diverse, the initial separation of the splanchnic plexus occurs over a length of not even 250 µm. Given our description of normal development of the pulmonary venous return from the cranial aspect of this splanchnic plexus, and the relation of the caudal part of the plexus with the developing portal vein, the variability of abnormal venous connections can be better appreciated, and the etiology of these types of malformations can be readily inferred (Figure 7), as has also been previously emphasized [42]. Drainage via the right common cardinal system represents failure of the separation of the normal pulmonary vein, and a communication of the splanchnic plexus with the right common cardinal vein. Drainage via the left common cardinal system follows a similar principle. Drainage into the umbilicovitelline system reflects failure to separate the normal pulmonary vein from the cranial aspect of the splanchnic plexus, with continuing drainage into the developing hepatic circulation.

### Evolutionary considerations of pulmonary venous development

**Figure 8:**
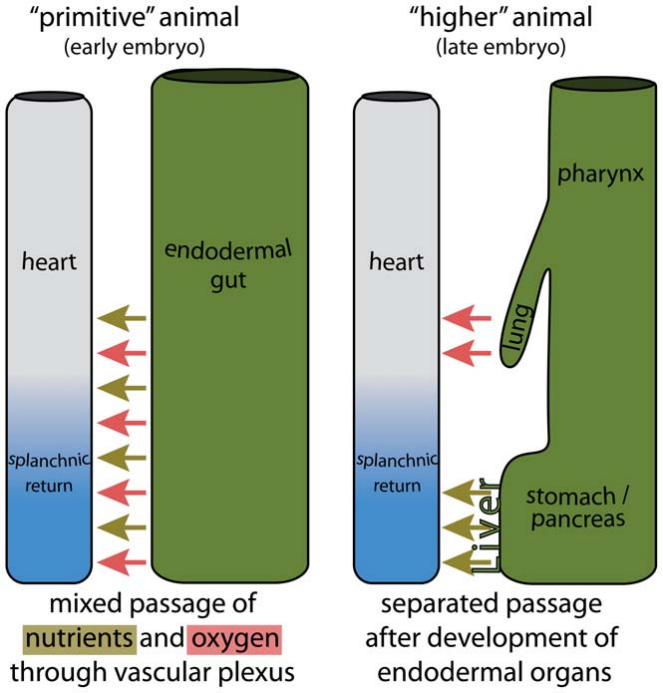
Simplification of phylogeny of splanchnic venous returns. The left panel illustrates a “primitive” animal, such as a mollusk. The endoderm is a simple tube. Overlaying this tube is a splanchnic vascular plexus, through which nutrients (green arrows) and oxygen (red arrows) pass and enter the vitelline venous system. These are then distributed throughout the body by a sluggishly contracting vessel, *i.e.* the heart. The right panel illustrates a transition that occurred in “higher” animals. Cranially in the endodermal tube, lungs have formed, which are specialized in the uptake and passage of oxygen. Caudally, specialized organs for the uptake and processing of nutrients, such as the stomach and pancreas, have formed. The flow of the afferent vessels of these structures has become separated.

The embryonic development of the pulmonary vein from a splanchnic plexus may also allow for some speculation on the evolutionary origins of the pulmonary circulation. Primitive animals resemble the earliest embryonic stages of “higher” vertebrates by the presence of an extensive vascular plexus that surrounds a tubular endodermal gut [43]. This gut does not contain specialized organs, and both oxygen and nutrients that pass through the gut are taken up via this venous plexus, to be distributed throughout the body by the peristaltic pumping of a simple unseptated heart (Figure 8).

As aerial ventilation via lungs arose, the heart gradually became septated, and the circulatory system evolved into a parallel arrangement of systemic and pulmonary blood flow. Essentially, the pulmonary vein separated from the venous returns of other endodermal organs, such as the stomach and the intestines. Evolution of incomplete septa, as still seen in present-day lungfish and amphibians [44], generated left and right sided atrial, ventricular, and outflow compartments that increased the efficiency of separation of the circulatory systems. In mammals and birds these flows have become entirely separated by formation of complete atrial and ventricular septa. During embryogenesis, however, our circulatory system conceptually recapitulates the evolution of our ancestors; starting with a simple straight cardiac tube that gradually septates into the four-chambered mammalian heart, receiving blood from two parallel circulations.

## Supporting Information

Figure S1 **Exemplary sections of a 2-day-old chicken embryo.** Above row shows a ventral and a dorsal view of the reconstructed heart and vessels, in relation to the displayed sections. Bottom three rows show sections that were segmented to generate the reconstruction; the left column shows *cTnI* stained section, the right column shows *Cx40* stained sections. (Abbreviations – cTnI: cardiac Troponin I; Cx40: Connexin40; OFT: outflow tract; * indicates the breakthrough of the dorsal mesocardium.)
(TIF)

Figure S2 **Exemplary sections of a 3-day-old chicken embryo.** Above row shows a ventral and a dorsal view of the reconstructed heart and vessels, in relation to the displayed sections. Bottom three rows show sections that were segmented to generate the reconstruction; the left column shows *cTnI* stained section, the right column shows *Cx40* stained sections. (Abbreviations – cTnI: cardiac Troponin I; Cx40: Connexin40; oft: outflow tract).
(TIF)

Figure S3 **Exemplary sections of a 4-day-old chicken embryo.** Above row shows a ventral and a dorsal view of the reconstructed heart and vessels, in relation to the displayed sections. Bottom three rows show sections that were segmented to generate the reconstruction; the left column shows *cTnI* stained section, the right column shows *Cx40* stained sections. (Abbreviations – cTnI: cardiac Troponin I; Cx40: Connexin40; av-canal: atrioventricular canal).
(TIF)

Figure S4 **Exemplary sections of a 5-day-old chicken embryo.** Above row shows a ventral and a dorsal view of the reconstructed heart and vessels, in relation to the displayed sections. Bottom three rows show sections that were segmented to generate the reconstruction; the left column shows *cTnI* stained section, the right column shows *Cx40* stained sections. (Abbreviations – cTnI: cardiac Troponin I; Cx40: Connexin40; av-canal: atrioventricular canal).
(TIF)

File S1 **Interactive 3D pdf.** This file offers interactive versions of the four reconstructions presented in this paper. For technical information refer to the first page of this pdf-file.
(PDF)
