# Supplementary figures and images for: Development of the Pulmonary Vein and the Systemic Venous Sinus: An Interactive 3D Overview
Source: PLoS One. 2011 Jul 11;6(7):e22055. doi: 10.1371/journal.pone.0022055 (PMC3133620; doi:10.1371/journal.pone.0022055)

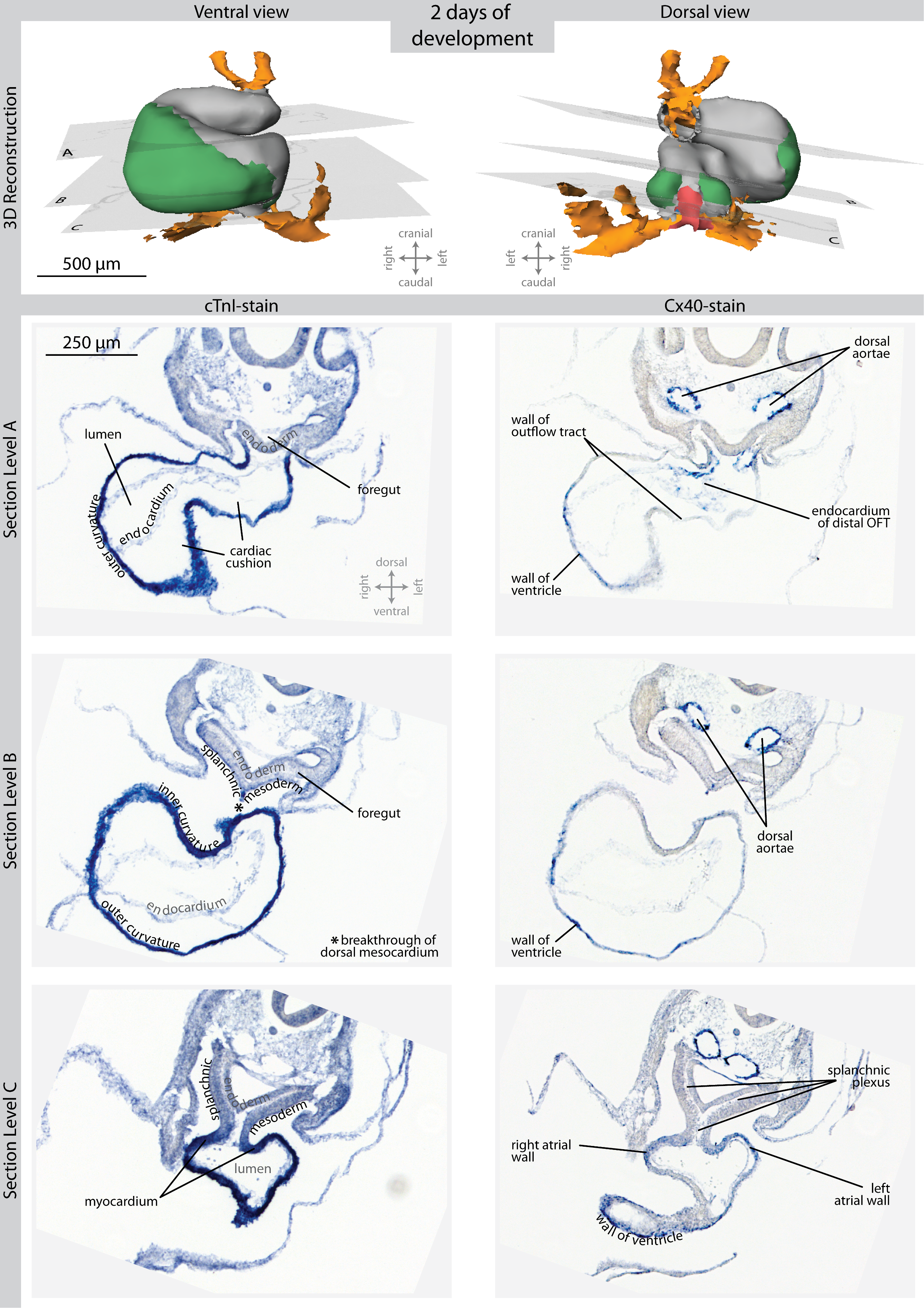

Supplement: Figure S1 — Exemplary sections of a 2-day-old chicken embryo. Above row shows a ventral and a dorsal view of the reconstructed heart and vessels, in relation to the displayed sections. Bottom three rows show sections that were segmented to generate the reconstruction; the left column shows cTnI stained section, the right column shows Cx40 stained sections. (Abbreviations – cTnI: cardiac Troponin I; Cx40: Connexin40; OFT: outflow tract; * indicates the breakthrough of the dorsal mesocardium.) (TIF) [file pone.0022055.s001.tif]

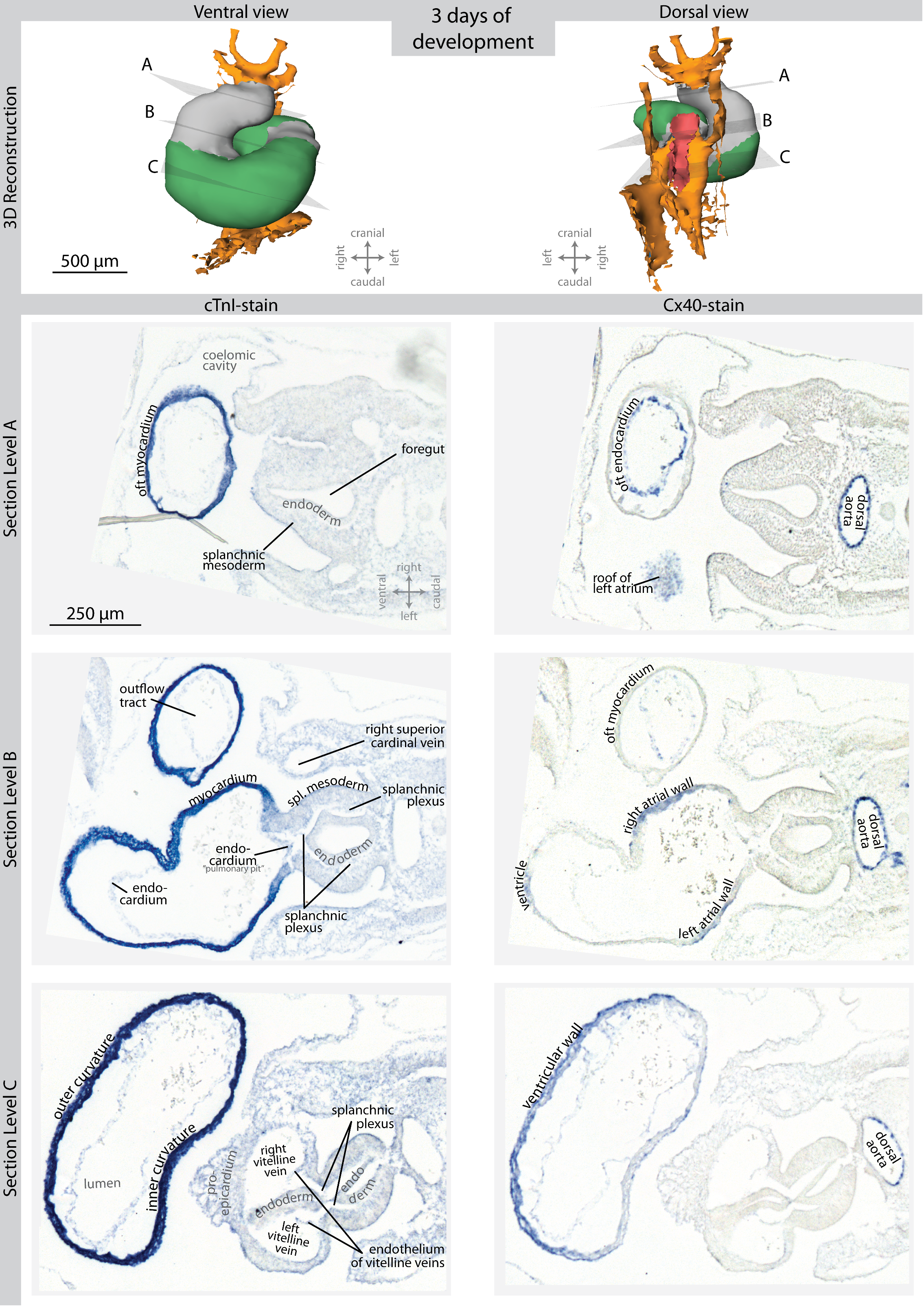

Supplement: Figure S2 — Exemplary sections of a 3-day-old chicken embryo. Above row shows a ventral and a dorsal view of the reconstructed heart and vessels, in relation to the displayed sections. Bottom three rows show sections that were segmented to generate the reconstruction; the left column shows cTnI stained section, the right column shows Cx40 stained sections. (Abbreviations – cTnI: cardiac Troponin I; Cx40: Connexin40; oft: outflow tract). (TIF) [file pone.0022055.s002.tif]

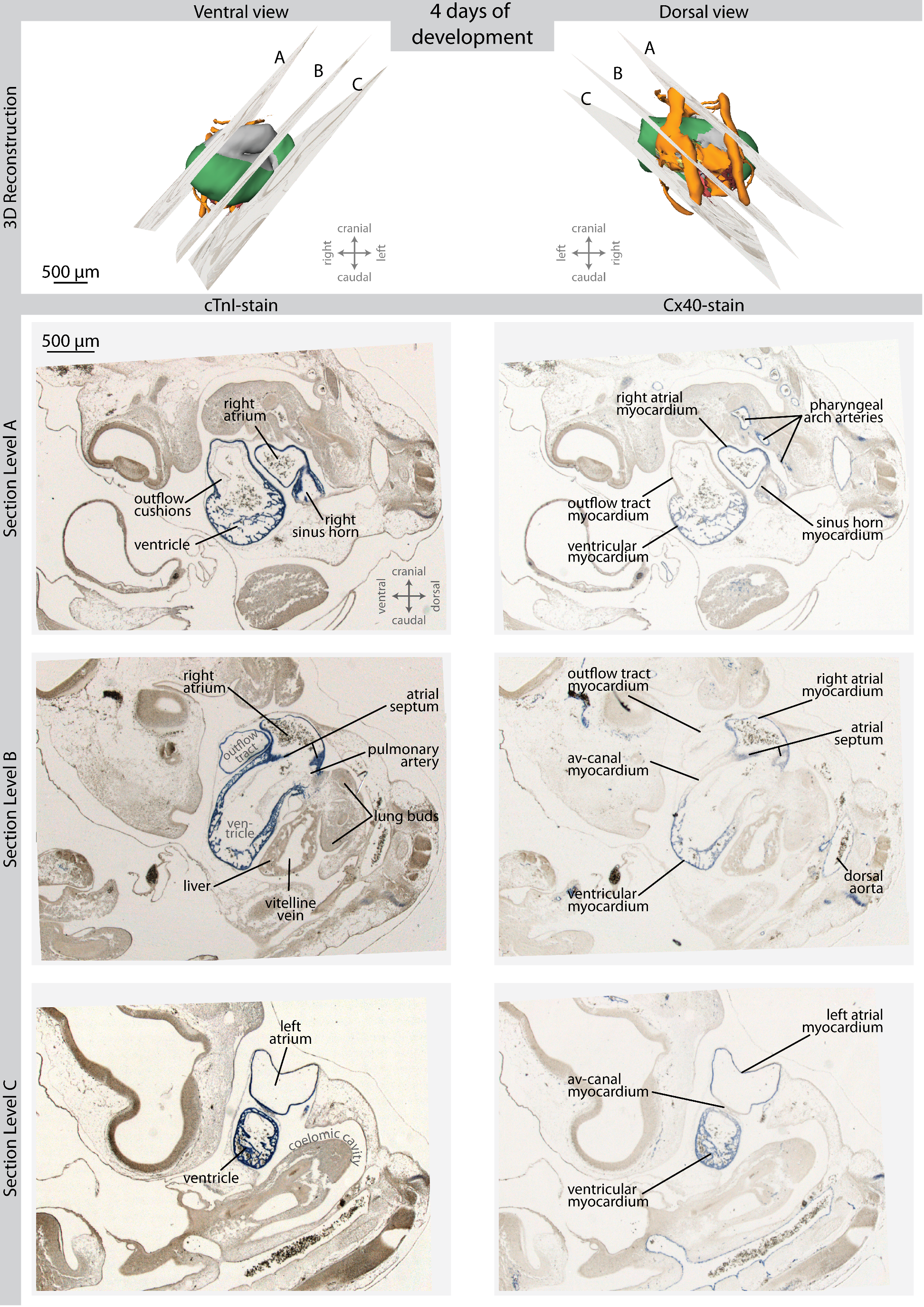

Supplement: Figure S3 — Exemplary sections of a 4-day-old chicken embryo. Above row shows a ventral and a dorsal view of the reconstructed heart and vessels, in relation to the displayed sections. Bottom three rows show sections that were segmented to generate the reconstruction; the left column shows cTnI stained section, the right column shows Cx40 stained sections. (Abbreviations – cTnI: cardiac Troponin I; Cx40: Connexin40; av-canal: atrioventricular canal). (TIF) [file pone.0022055.s003.tif]

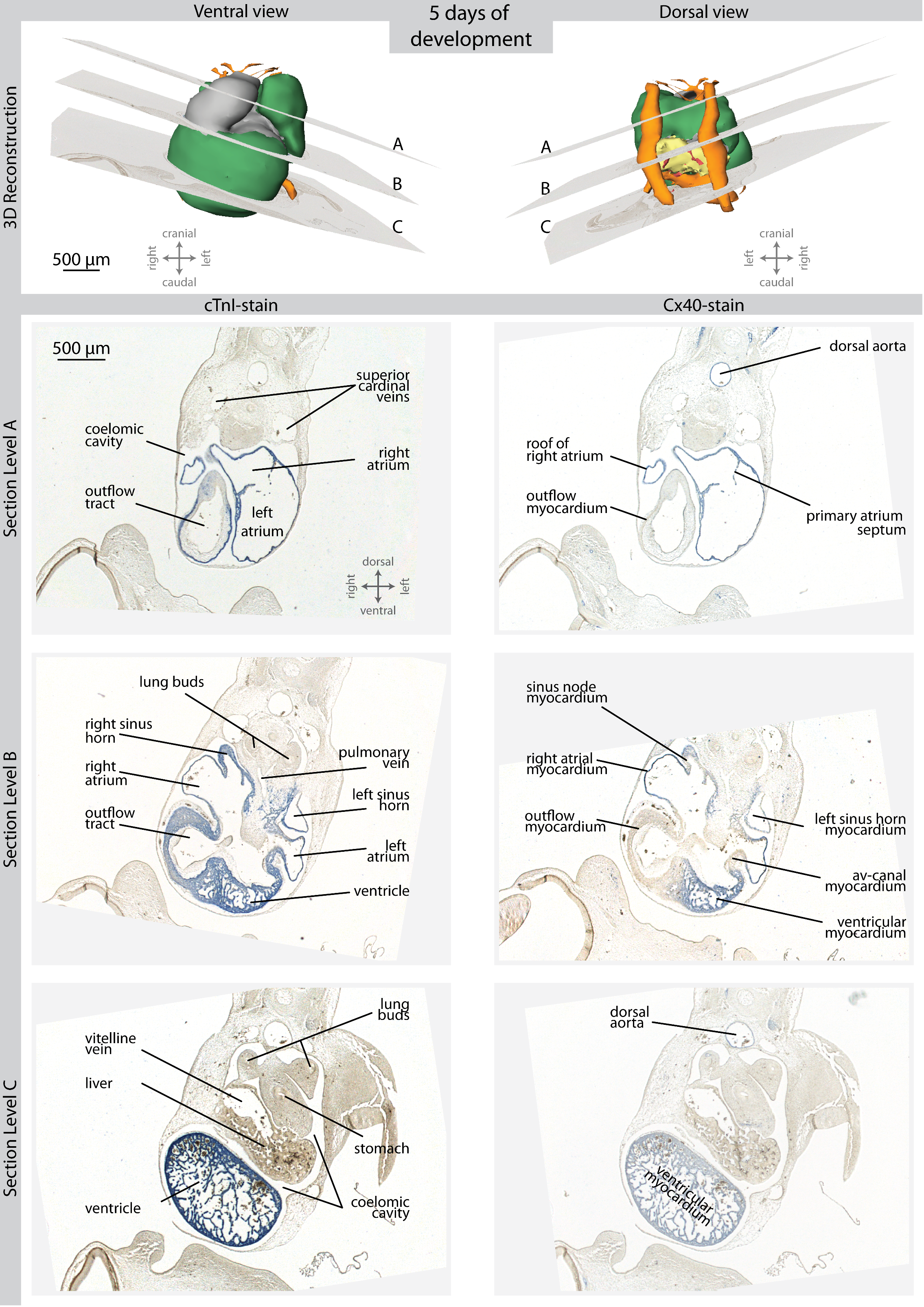

Supplement: Figure S4 — Exemplary sections of a 5-day-old chicken embryo. Above row shows a ventral and a dorsal view of the reconstructed heart and vessels, in relation to the displayed sections. Bottom three rows show sections that were segmented to generate the reconstruction; the left column shows cTnI stained section, the right column shows Cx40 stained sections. (Abbreviations – cTnI: cardiac Troponin I; Cx40: Connexin40; av-canal: atrioventricular canal). (TIF) [file pone.0022055.s004.tif]
