# Supplementary material for: Development of the Pulmonary Vein and the Systemic Venous Sinus: An Interactive 3D Overview
Source: PLoS One. 2011 Jul 11;6(7):e22055. doi: 10.1371/journal.pone.0022055 (PMC3133620; doi:10.1371/journal.pone.0022055)
Supplement: File S1 — Interactive 3D pdf. This file offers interactive versions of the four reconstructions presented in this paper. For technical information refer to the first page of this pdf-file. (PDF) [file pone.0022055.s005.pdf]

Information about the use of this interactive 3D-pdf

Inflow
- Myocardium Cx40 –
- Mesenchyme
- Umbilicovitelline vein
- Left cardinal vein
- Right cardinal vein
- Hepatic Lumen
- Liver

Atrium
- Myocardium Cx40 –
- Myocardium Cx40 +
- Mesenchyme
- Lumen

Outflow
- Myocardium Cx40 –
- Myocardium Cx40 +
- Mesenchyme
- Lumen

Various
- Splanchnic plexus / Pulmonary vein
- Endoderm
- Splanchnic Mesoderm
- Coelom
- Somatic Mesoderm

Heart Failure Research Center
http://3d.hfrc.nl

To interact with the reconstructions

Rotate: Hold left mouse-button and move mouse.

Zoom: Hold right mouse-button and move mouse up or down.

Translate: Hold left and right mouse-buttons and move mouse.

Selection of structures

The left panel contains buttons for each structure to either show, hide, or make transparent.

For the the oldest stages, there are also buttons to collectively show or hide cardiac compartments (inflow, atrium, outflow, and various).

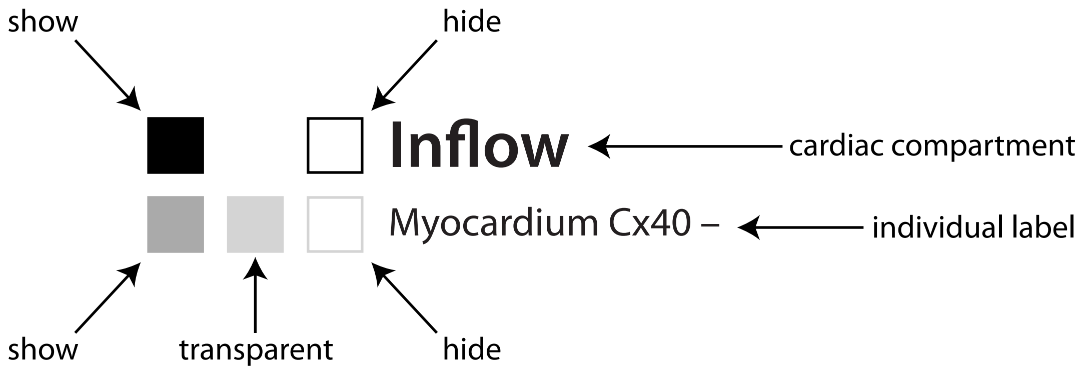

Selection of preset views

The bottom panel contains several preset views. Click to select such a view.

Link to our website

Click on AMC-logo to visit our website.

Technical Notes
This PDF file is preferably viewed in Adobe Reader® 9.3 or higher (http://www.adobe.com/downloads/)
Javascript must be enabled.

Open *Edit* ⇨ *Preferences* to ensure the following:
1) In *3D& Multimedia*, under *3D Tool Options*
   - for *Open Model Tree on 3D Activation* choose *Use Annotation's Settings*
   - for *Default Toolbar State* choose *Use Annotation's Settings*
   - disable *Show 3D Orientation Axis*
2) In *3D& Multimedia*, under *Auto-Degrade Options*
   - for *Optimalization Scheme for Low Framerate* select *None*
3) In *JavaScript*, under *JavaScript*
   - enable *Enable Acrobat JavaScript*

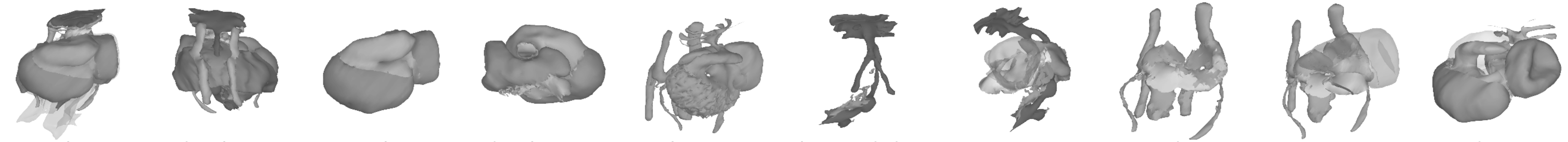

ventral view | dorsal view | ventral myo. | dorsal myo. | lumen | plexus/endoderm | PV/LA | sinus horns | systemic/RA | working myo.

Interactive Reconstruction of 2-day-old Chicken Embryo

Intracardiac

- Myocardium Cx40 –
- Myocardium Cx40 +
- Cardiac Cushion
- Lumen

Extracardiac

- Splanchnic mesoderm
- Splanchnic plexus
- Coelom
- Somatic mesoderm
- Endoderm

Lumen

- Vitelline Veins
- Left Cardinal Vein
- Right Cardinal Vein

Heart Failure
Research Center
http://3d.hfrc.nl

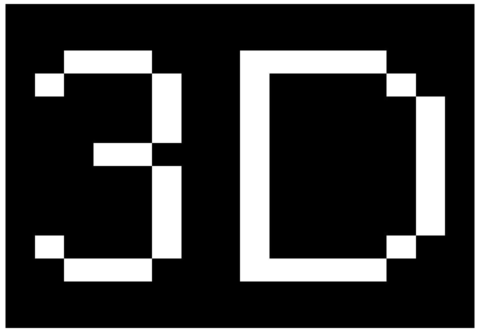

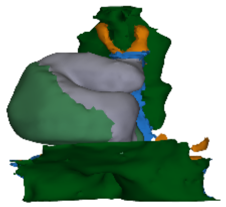

frontal view

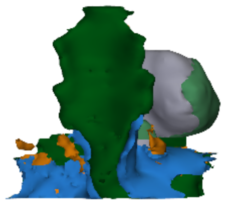

dorsal view

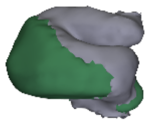

frontal - myo

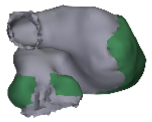

dorsal - myo

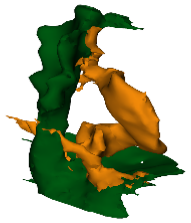

foregut/lumen

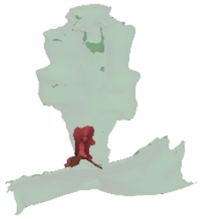

foregut/plexus

Interactive Reconstruction of 3-day-old Chicken Embryo

Intracardiac

- Myocardium Cx40 –
- Myocardium Cx40 +
- Cardiac Cushion
- Lumen

Extracardiac

- Splanchnic mesoderm
- Splanchnic plexus
- Coelom
- Somatic mesoderm
- Endoderm

Lumen

- Vitelline Veins
- Left Cardinal Vein
- Right Cardinal Vein

Heart Failure
Research Center
http://3d.hfrc.nl

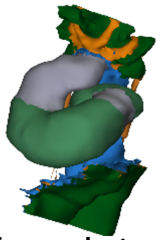
frontal view

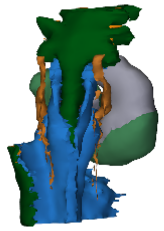
dorsal view

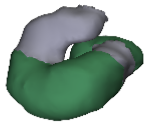
frontal - myo

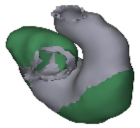
dorsal - myo

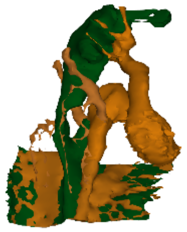
foregut/lumen

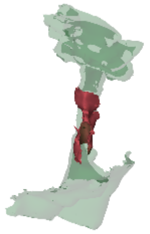
foregut/plexus

Interactive Reconstruction of 4-day-old Chicken Embryo

Inflow

- Myocardium Cx40 –
- Mesenchyme
- Umbilicovitelline vein
- Left cardinal vein
- Right cardinal vein
- Hepatic Lumen
- Liver

Atrium

- Myocardium Cx40 –
- Myocardium Cx40 +
- Mesenchyme
- Lumen

Odr-tcI rpgajc

- Myocardium Cx40 –
- Myocardium Cx40 +
- Mesenchyme
- Lumen

Various

- Splanchnic plexus / Pulmonary vein
- Endoderm
- Splanchnic Mesoderm
- Coelom
- Somatic Mesoderm

Heart Failure
Research Center
http://3d.hfrc.nl

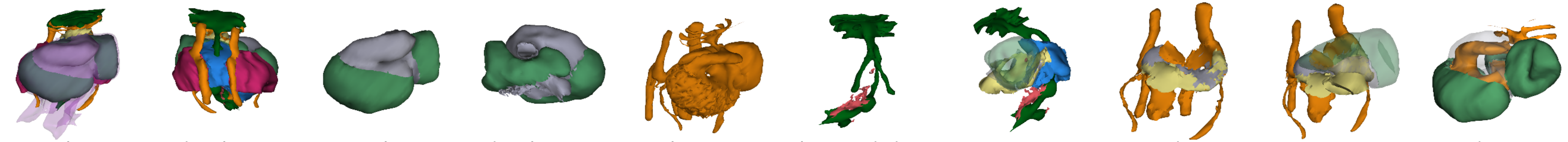

ventral view | dorsal view | ventral myo. | dorsal myo. | lumen | plexus/endoderm | PV/LA | sinus horns | systemic/RA | working myo.

Interactive Reconstruction of 5-day-old Chicken Embryo

Inflow

- Myocardium Cx40 –
- Myocardium Cx40 +
- Umbilicovitelline vein
- Left cardinal vein
- Right cardinal vein
- Hepatic Lumen
- Mesenchyme
- Base of epicardium

Atrium

- Myocardium Cx40 –
- Myocardium Cx40 +
- Mesenchyme
- Lumen

Odr-tc I rpgajc

- Myocardium Cx40 –
- Myocardium Cx40 +
- Mesenchyme
- Lumen

Various

- Splanchnic plexus / Pulmonary vein
- Endoderm
- Coelom

Heart Failure
Research Center
http://3d.hfrc.nl

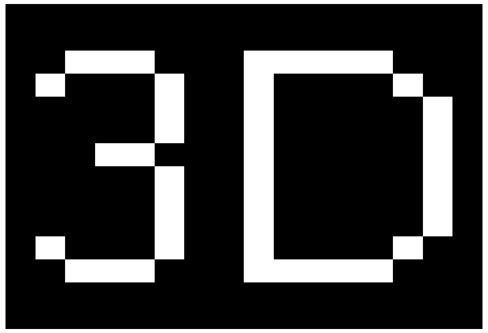

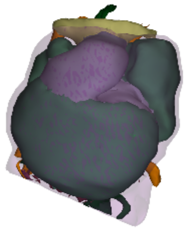
ventral view

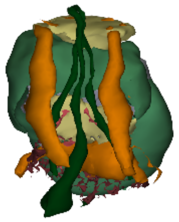
dorsal view

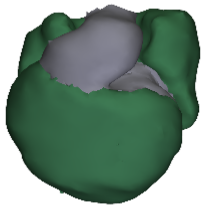
ventral myo.

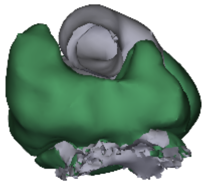
dorsal myo.

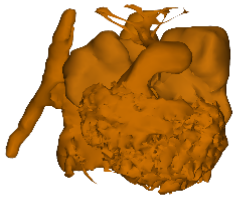
lumen

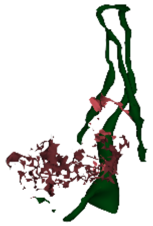
plexus/endoderm

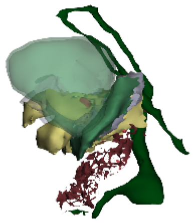
PV/LA

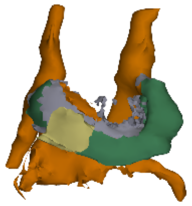
sinus horns

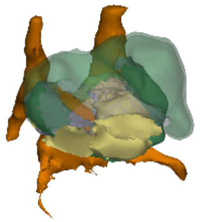
systemic/RA

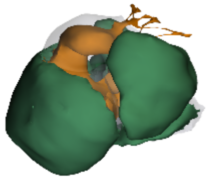
working myo.
